# Supplementary material for: Expression, intracellular localization, and mutation of EGFR in conjunctival squamous cell carcinoma and the association with prognosis and treatment
Source: PLoS One. 2020 Aug 24;15(8):e0238120. doi: 10.1371/journal.pone.0238120 (PMC7444806; doi:10.1371/journal.pone.0238120)
Supplement: Attachment — Submitted filename: Plos One review.docx [file pone.0238120.s002.docx]

In the submitted manuscript, Sakai *et al* evaluated the expression, localization, and mutation of EGFR in conjunctival squamous cell carcinoma. Using immunohistochemistry, they observed that all 29 samples were EGFR positive and classified its expression into membrane, cytoplasm, focal, and diffuse. Eight samples presented the E746-A750 del mutation while 2 samples the L858R point mutation. They also found a correlation between EGFR cytoplasmic staining and orbital exenteration, and between decreased EGFR membrane staining and progression-free survival. Although, the role of EGFR has been extensively study in many different cancer types, little is known about EGFR in conjunctival squamous cell carcinoma. The current study brings valuable information to the field and is on a topic of relevance. However, some concerns must be addressed before further consideration.

Specific comments

1. The result section should be written in more detail for easier reading.

2. Line 154: “Analysis of EGFR intracellular staining patterns showed scores of 1.72 for membrane staining and 1.48 for cytoplasmic staining (Figure 1)”. This result is found in Table 2 not in figure 1.

3. Table 2: Revise statistics. My calculation doesn’t match the average shown in the table.

4. Is there any difference in EGFR expression localization (membrane or cytoplasm) between focal and diffuse staining?

5. Line 156: “Seven benign conjunctival lesions were partially weak positive in conjunctival squamous epithelial cells near basement membrane”. Specify the total number of benign samples evaluated (7 out of 7?). It would be interesting to show the score analysis of benign samples to compare to tumor samples.

6. Line 157: “We found no significant changes between Tis (in situ) and Tadv (SCC advanced cases) (Table 2)”. Specify what changes.

7. Line 159: Include more information regarding EGFR E746-A750 del and EGFR L858R expression like diffuse/focal staining, membrane/cytoplasmic score. Is there any correlation between EGFR mutation and clinical data (orbital exenteration/PFS)?

8. Table 1: EGFR expression in tumors - diffuse staining (8) and focal staining (19). Are these numbers correct, total samples 27?

9. Figure 1 and 2: When comparing images use the same magnification. Verify if scale bars are correct.
