# Supplementary material for: Expression, intracellular localization, and mutation of EGFR in conjunctival squamous cell carcinoma and the association with prognosis and treatment
Source: PLoS One. 2020 Aug 24;15(8):e0238120. doi: 10.1371/journal.pone.0238120 (PMC7444806; doi:10.1371/journal.pone.0238120)
Supplement: Attachment — Submitted filename: Response to reviewers R2.docx [file pone.0238120.s003.docx]

Response to reviewers

Reviewer #1: Thank you for your valuable comments. We submitted the revised version for proofreading by a native English-speaking science editor.

Reviewer #3

Thank you for your valuable comments. Our responses are below.

1. A major revision of language and grammar is necessary for any further consideration. There are several typos, missing spaces between words, and grammatically incorrect sentences (especially in the new text added after revision) that make it hard to read. Some examples are:
Line 161 - Benign cases showed scores of 1.28 for membrane staining and 0.14 for cytoplasmic staining, compared with conjunctival Scc cases, Cytoplasmic staining was statistically significant changes
Line 166 - The correlation between EGFR stating (focal or diffuse) and EGFR localization (membrane group) was no significant difference and tendency
Line 180 - COX regression model was used to examine and analysis among clinicopathological status, EGFR staining pattern, and EGFR mutation for long-term prognosis including orbital exenteration and.PFS.
Line 188 - In addition, the EGFR 188 mutation was not significantly correlation with final orbital exenteration and PFS(Table 4 and 5).

We submitted the revised version for proofreading by a native English-speaking science editor.

2. Line 206 – “There was no significant difference in this study”. Specify what had no significant difference. The whole study or a specific result?

Only the results of this statement are mentioned. We corrected the sentence as shown below.

L170“Intracellular transfer of EGFR in the group with diffuse staining may indicate progression, and although no statistical differences were observed in this study, significant findings may emerge by increasing the number of cases in the future.”

3. Line 160, the sentence “EGFR expression in colon cancer for positive control” is out of context. What is the relevance of this information to the results?
We corrected the sentence as shown below. The location was changed to line 163 to suit the context. Accordingly, we changed the location of the Figure 2 citation in the text.

4. Line 164-167 – Regarding the correlation between EGFR staining (focal or diffuse) and EGFR localization (membrane or cytoplasm), it would be interesting to have a table showing the number of diffuse and focal staining samples in the membrane, and cytoplasm group. Between the slides with EGFR membrane localization, how many samples had focal staining and how many had diffuse staining? Add the same information for EGFR cytoplasm localization.

We created a new Table 3 to address your comment.
**Table3. EGFR staining and Localization**

|  | EGFR Focal(N=21) | EGFR Diffuse（N=8) | *P* |
| --- | --- | --- | --- |
| Cell Membrane | 1.8±0.9 | 1.5±0.9 | 0.38 |
| Cell cytoplasmic | 1.3±0.6 | 1.8±0.8 | 0.12 |

p value based on the non-paired t-test.

5. In table 3, add information (number of samples) regarding focal, diffuse, membrane, and cytoplasmic staining for each mutation.

We created a new Table 4 to hold this information.

**Table 4. Summary of *EGFR* E746-A750 del and *EGFR* L858R point mutations**

| Mutation | N = 29 (%) | Age (y) | Sex (M/F) | T stage | EGFR staining patterns (diffuse/focal) | EGFR localization score (membrane/cytoplasmic) |
| --- | --- | --- | --- | --- | --- | --- |
| Exon 19 *EGFR* E746-A750 del (N=8) | 8/29 (27.5%) | 75.8 | 3/5 | T3: 4  T2: 2  Tis: 2 | 2/6 | 1.6/1.5 |
| Exon 21 *EGFR* L858R point mutation (N=3) | 2/29 (6.8%) | 73.0 | 1/1 | T3: 1  Tis: 1 | 1/2 | 1/3 |

M: Male, F: Female

6. In figure 3C, the EGFR L858R staining seems to be localized in the nucleus. Do you have an explanation for that?

Thank you for this advice. We agree that regarding the dyeability of EGFR, it is important whether or not the cell membrane is stained. It looks like it stains the nucleus, but we are unsure of the exact reason. We believe that it differs from the simple background because only the nuclei of squamous cell carcinoma are stained.

There are the following documents. Whether or not this is meant is not convinced by this paper alone.

The receptor tyrosine kinases (RTK) of the epidermal growth factor receptor (EGFR) superfamily　in the membrane can translocate to the nucleus through different mechanisms. Nuclear RTKs regulate a variety of cellular functions, such as cell proliferation, DNA damage repair, and signal transduction, both in normal tissues and in human cancer cell. In addition, they play important roles in determining cancer response to cancer therapy.（Shao-Chun Wang et al. **DOI:** 10.1158/1078-0432.CCR-08-2813  2009）
